# Laparoscopic Kasai portoenterostomy for biliary atresia: first experience from Central Asia

**DOI:** 10.3389/fped.2025.1666539

**Published:** 2025-10-03

**Authors:** Konstantin Semash, Mansur Nasirov, Timur Dzhanbekov, Ayimgul Khudaybergenova

**Affiliations:** Department of Minimally Invasive Surgery and Transplantation, National Children's Medical Center, Tashkent, Uzbekistan

**Keywords:** laparoscopic Kasai portoenterostomy, biliary atresia, minimally invasive surgery, pediatric surgery, liver transplantation

## Abstract

**Objective:**

Biliary atresia (BA) is a progressive fibro-obliterative disease of the extrahepatic bile ducts. Laparoscopic Kasai portoenterostomy (LKPE) has emerged as a minimally invasive alternative to the open approach. We aimed to evaluate the feasibility, perioperative outcomes, and early prognostic factors of LKPE in a single center.

**Methods:**

We conducted a retrospective single-center study. Feasibility, operative metrics (including the learning curve), incidence of cholangitis, native liver survival, and clinical outcomes were assessed using standard statistical methods.

**Results:**

Among the 33 patients (14 females, 19 males), the age at surgery was <60 days (12 patients), 60–89 days (13 patients), and >90 days (8 patients). The median operative time was 240 min, showing a declining trend with increasing experience. One conversion (1/33) to open surgery was required due to inadequate hilar visualization. Postoperative cholangitis occurred in 51.5% of cases and was associated with an increased risk of native liver failure (HR = 3.6, *p* = 0.051). The native liver survival rate at study completion was 54.5%, with 5 patients (15.2%) requiring liver transplantation. The overall mortality rate was 30.3%, primarily due to sepsis and cholangitis-related complications.

**Conclusions:**

LKPE is a feasible and effective surgical option for biliary atresia. Despite its advantages, including enhanced hilar visualization and faster recovery, the high incidence of postoperative cholangitis remains a major challenge that adversely affects native liver survival. Optimizing perioperative management, implementing prophylactic strategies against cholangitis, and expanding pediatric liver transplantation programs in resource-limited settings are essential to improving outcomes. Further prospective studies with long-term follow-up are needed to refine surgical techniques and optimize patient management.

## Highlights

This study presents the first experience with laparoscopic Kasai portoenterostomy (LKPE) for biliary atresia (BA) in Central Asia. Conducted at a single center in Uzbekistan, where pediatric liver transplantation is not yet available, the study demonstrates that LKPE is a feasible and effective surgical option. It outlines key technical adaptations, evaluates the learning curve, and highlights postoperative cholangitis as a major prognostic factor for native liver survival. The findings emphasize the critical role of LKPE as the only life-saving intervention currently accessible to infants with BA in resource-limited settings.

## Introduction

Biliary atresia (BA) is a progressive, fibro-obliterative disease of the extrahepatic bile ducts, leading to obstructive cholestasis, fibrosis, and ultimately, liver failure if left untreated. It is the most common cause of neonatal cholestasis and a leading indication for pediatric liver transplantation worldwide (1). The gold-standard surgical intervention for BA is the Kasai portoenterostomy (KPE), which aims to restore bile flow by creating a direct anastomosis between the porta hepatis and a Roux-en-Y jejunal limb (2). Successful KPE can significantly delay or, in some cases, even prevent the need for liver transplantation (3). However, its outcomes are highly dependent on early diagnosis, surgical expertise, and postoperative management.

Traditionally, open KPE has been the standard approach. However, over the past two decades, there has been growing interest in the laparoscopic Kasai portoenterostomy (LKPE) as a minimally invasive alternative (4–7). LKPE offers several advantages, including reduced postoperative pain, shorter hospital stays, improved cosmesis, and enhanced visualization of biliary structures through magnified laparoscopy (7). Despite these benefits, its adoption has been limited by concerns about its technical complexity, prolonged operative time, and a perceived learning curve that may affect early outcomes (7–9).

One of the main challenges after KPE, open or laparoscopic, is postoperative cholangitis, a major cause of disease progression and loss of native liver (10). Cholangitis, often triggered by ascending bacterial infections, contributes to ongoing hepatic fibrosis, leading to cirrhosis and the eventual need for liver transplantation (11). Understanding the impact of cholangitis and optimizing strategies for its prevention and management are critical for improving long-term outcomes in BA patients.

The Kasai procedure is recognized as a bridge to liver transplantation in children with biliary atresia, as it helps stabilize the child's condition and allows for necessary weight gain, which directly influences liver transplantation outcomes (12). In Uzbekistan, the estimated annual demand for surgical intervention for biliary atresia, including Kasai portoenterostomy or liver transplantation, is approximately 60–80 patients, corresponding to 1.6–2.1 cases per 100 000 population. The system enabling early diagnosis is underdeveloped, and there is no unified national screening protocol. As a result, many children are referred too late for Kasai portoenterostomy, which lowers the chances of keeping their native liver. Uzbekistan is a developing country in Central Asia, where a liver transplantation program for infants is not yet established. A liver transplantation program for adults was recently initiated in the country (13). In December 2024, our center successfully transplanted the first liver in an adolescent child (14). However, there is currently no expertise in the country to perform liver transplantation for infants with biliary atresia. At present, our center is actively training specialists to launch a liver transplantation program for infants with biliary atresia. Nevertheless, children with biliary atresia in Uzbekistan face a dire prognosis. Many families lack the financial means to seek liver transplantation abroad. The Kasai procedure remains the only lifeline for these children, offering them a chance for survival.

This study presents a single-center analysis of 33 initial cases of LKPE in Uzbekistan, evaluating its feasibility, surgical outcomes, learning curve, incidence of cholangitis, and native liver survival rates. We also assess the learning curve associated with LKPE and discuss key factors influencing its success, including technical refinements, perioperative care protocols, and the role of adjunctive therapies, such as steroids and antibiotics. To the best of our knowledge, this is the first systematic evaluation of laparoscopic Kasai portoenterostomy in Uzbekistan, a country without an established pediatric liver transplantation program. Our findings provide a crucial perspective on the feasibility of LKPE as the only available surgical option for BA in resource-limited settings. By sharing our experience, we aim to contribute to the ongoing discussion about the potential of LKPE as a viable surgical option for BA and identify areas for further improvement in its clinical application.

## Methods

### Study design and setting

This study employed a retrospective design to analyze outcomes in 33 consecutive cases of biliary atresia treated with LKPE at our center from July 2022 to January 2025. Open Kasai procedures were not conducted at our center by our team. Historically, approximately ten open Kasai procedures had been performed at our center by a different surgical team; however, no systematic database existed for these cases. Furthermore, some procedures had been carried out without clear indications, such as in patients with Alagille syndrome, and these were excluded to ensure diagnostic accuracy and consistency of the dataset.

Patient data, including demographic details, clinical presentation, surgical interventions, and follow-up outcomes, were collected from electronic medical records and analyzed. Inclusion criteria consisted of patients diagnosed with biliary atresia based on clinical, biochemical, and radiological findings consistent with the Japanese Association for Biliary Atresia guidelines (3). Exclusion criteria included patients who underwent open Kasai portoenterostomy or had incomplete medical records.

The study was approved by the Institutional Review Board of the National Children's Medical Center, Tashkent, Uzbekistan (IRB statement #717-66-2025). The patients provided written consent allowing the use of medical data for scientific research while ensuring the anonymity of the patients.

### Statistical analysis

Statistical analysis was performed using SPSS version 26, Jamovi software, version 2.3.28.0, and G*Power version 3.1.9.6. The normality of the distribution of quantitative variables was assessed using the Shapiro–Wilk test. For descriptive statistics, continuous variables are presented as medians with ranges, while categorical variables are presented as absolute values and percentages. Group comparisons for quantitative data were conducted using analysis of variance (ANOVA) for normally distributed variables. Categorical variables were compared using Pearson's chi-square test. Native liver survival and overall survival were evaluated using the Kaplan–Meier method, with differences between groups analyzed using the log-rank test. A multivariable logistic regression model was constructed to identify independent predictors of native liver survival. Additionally, Cox regression was used to assess the impact of cholangitis on native liver survival, allowing for the consideration of censored data and the estimation of the probability of native liver preservation over time. Linear regression was applied to analyze the learning curve patterns, enabling the evaluation of the reduction in operative time with increasing surgical experience. *post hoc* power analysis was performed for the logistic regression model to assess the adequacy of sample size and detectability of the association between postoperative cholangitis and native liver survival. The learning curve was assessed using a CUSUM (Cumulative Sum Control Chart) based on operative time. “Success” was defined as an operative time equal to or shorter than the mean operative time, while “failure” was defined as an operative time exceeding the mean. The CUSUM curve was plotted sequentially to visualize performance trends over time. A risk-adjusted CUSUM analysis was also conducted, incorporating patient-specific risk factors (Age, Sex, BA type, Bilirubin, GGT) to adjust expected performance benchmarks. A critical significance level of *p* < 0.05 was applied for all tests.

## Results

### Baseline characteristics

Since 2022, a total of 33 patients underwent the laparoscopic Kasai procedure, comprising 14 females (42.4%) and 19 males (57.6%). The age distribution at the time of surgery was as follows: less than 60 days (12 patients, 36.4%), between 60 and 89 days (13 patients, 39.4%), and greater than 90 days (8 patients, 24.2%). Clinical features commonly observed among the patients included prolonged jaundice, acholic stool defined as saturation less than 44 on the Japanese stool card, elevated levels of conjugated bilirubin, and a non-contractile gallbladder identified on abdominal ultrasound. Some patients underwent MRCP at their place of residence; however, these data were not used as criteria for diagnosing biliary atresia. All perioperative characteristics of patients are represented in Table 1.

**Table 1:** Patient characteristics and outcomes.

Patient characteristics	Age under 60 days	Age 60–89 days	Age more than 90 days	Total	*p*, value
Patients, *n*	12	13	8	33	
Age, days, median (range)	54.5 (27–58)	70 (60–89)	99 (90–148)	69 (27–148)	<0.001
Sex					0.105
Male	8	9	2	19	
Female	4	4	6	14	
BA type, n					0.246
Type I			1	1	
Type IIa		1		1	
Type IIb		1	1	2	
Type III	12	11	6	29	
T. bil at submision,	183 (99–314)	162 (121–298)	158 (95–376)	175 (95–376)	0.878
GGT at submission	335 (59–1,560)	504 (109–1,007)	743 (166–1,602)	504 (59–1,602)	0.413
Operation time, min	222.5 (120–570)	230 (115–425)	262.5 (180–360)	240 (115–570)	0.785
Conversion to open, *n* (%)	0	1	0	1 (3%)	0.478
LOS	11 (5–19)	15.5 (8–20)	15 (9–20)	14 (5–20)	0.584
Cholangitis after LAPKPE, *n* (%)	5	8	4	17 (51.5%)	0.631
LT after LAPKPE, m (%)	2	1	2	5 (15.2%)	0.577
Native liver survival	7	8	3	18 (54.5%)	0.557
Mortality, %	25	23	50	30.3	0.5
Follow up NLS, month, average (range)	6.7 (2–30)	7.8 (0–20)	7.5 (2–14)	7.3 (1–30)	0.894
Follow up, month, average (range)	6.7 (1–30)	6 (0–20)	7.5 (2–14)	9 (1–30)	0.849
Cause of death					0.224
GIT bleeding			1	1	
Aspiration			1	1	
Sepsis	2	1	1	4	
Acute renal failure			1	1	
MODS	1			1	
Unknown		2		2	

*n*, number; BA, biliary atresia; T.bil, total bilirubin; GGT, gamma-glutamyl transferase; min, minutes; LOS, length of stay; LT, liver transplantation; NLS, native liver survival; GI, gastro-intestinal; MODS, multiple organ dysfunction syndrome.

### Surgical technique

**Figure 1:**
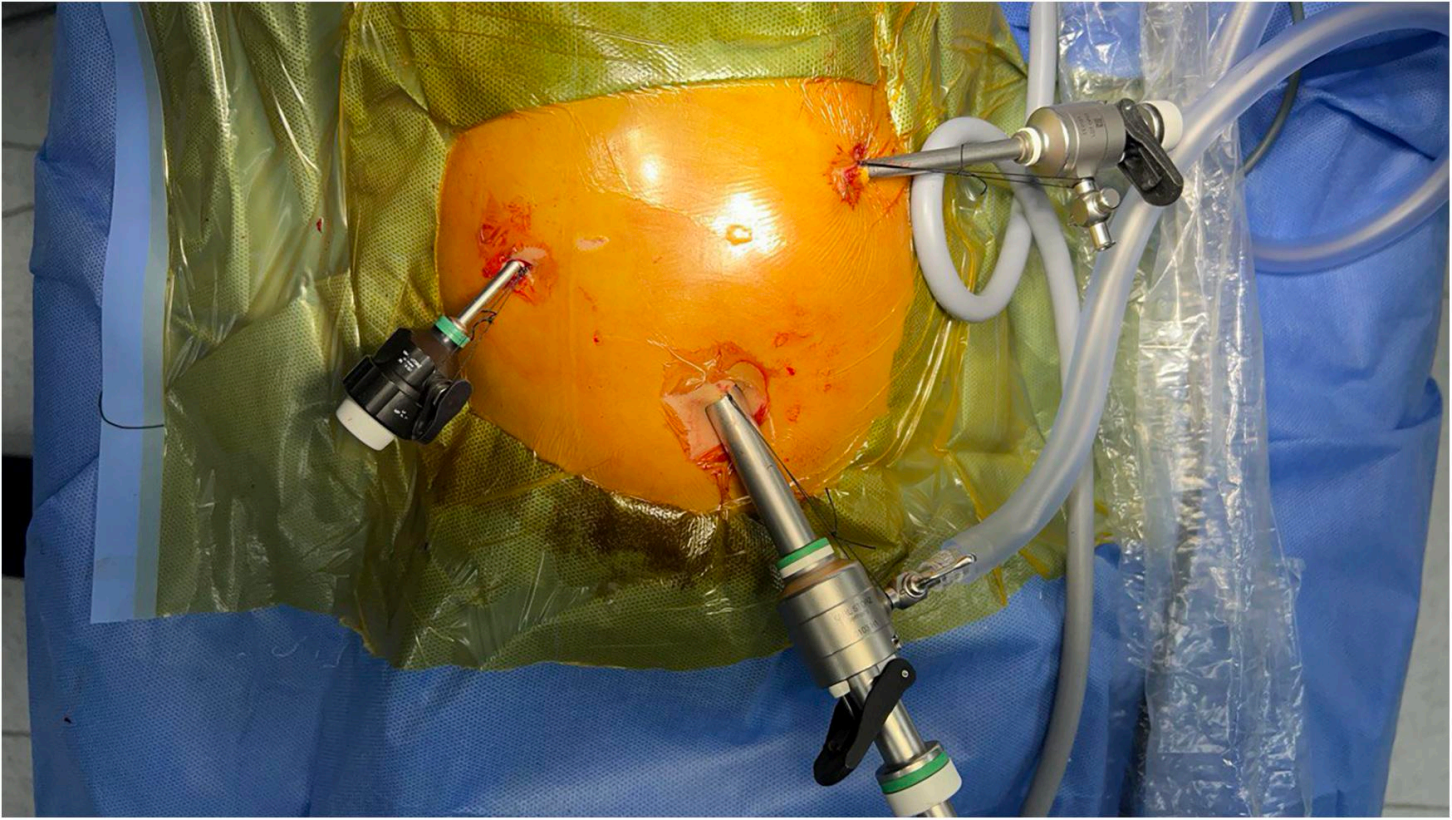
Port placement for laparoscopic Kasai portoenterostomy. The camera port (10 mm) is inserted through the umbilicus. Additional working ports (5 mm) are positioned in the right and left upper quadrants. Pneumoperitoneum is established through the umbilical port. This setup provides optimal visualization and ergonomic instrument manipulation during laparoscopic Kasai portoenterostomy.

The patient is positioned on the operating table in a head-up (reverse Trendelenburg) position. The procedure begins with the transumbilical placement of a 3 mm port and the introduction of a 30-degree, 3 mm camera. The intra-abdominal PCO_2_ pressure is set to 10–12 mmHg with an insufflation flow rate of 6 liters per minute. A thorough examination of the liver is performed. Subsequently, a 3 mm port is placed in the right abdominal quadrant for liver traction. A percutaneous cannula is then inserted, followed by cholangiography. If the diagnosis of BA is confirmed, prior to proceeding with the main surgical steps, the 3 mm umbilical port is replaced with a 10 mm port along with a camera change to enhance visualization. Additionally, a 5 mm port with a 3 mm instrument adapter is placed on the left side. Initially, a 3 mm right-sided port was used; however, we later adopted a 5 mm port to facilitate needle insertion and removal, which contributed to a reduction in operative time. Port placement is illustrated in Figure 1.

The next step involves the excision of atretic biliary structures and dissection of the hepatic hilum. The procedure begins with a laparoscopic cholecystectomy using a 3 mm bipolar instrument, with electrocautery applied until the cystic artery is visualized. At this stage, further dissection is performed using a blunt technique without energy application. The rudimentary hepatic ducts are subsequently mobilized. The hepatic arteries are identified at the level of the cystic artery and serve as the inferior boundary of the hepatic hilum. In all cases, the fibrotic bands meticulously dissected and completely excised. Traction was applied to the fibrotic plate to expose its base, and the bands were dissected using a combination of sharp and blunt techniques with scissors, without the use of energy devices. If oozing occurred, a gauze swab was applied with gentle pressure for 5–7 min until hemostasis was achieved. In addition, transient elevation of pneumoperitoneum to 12–14 mmHg was used to facilitate hemostasis and improve visualization. This approach allowed for adequate dissection of the fibrotic bands while minimizing the risk of bleeding. The hepatic hilum is then packed with gauze, and then the creation of a Roux-en-Y loop via the transumbilical approach is performed.

The instrument initially used for traction of segment IVb is subsequently utilized for retracting the transverse colon and identifying Treitz's ligament. The small intestine is measured 15–20 cm distally and secured using a 5-mm intestinal grasper, with the instrument tip oriented distally for reference. Intraperitoneal marking is not utilized as we do not find it necessary. The umbilical port is then removed, and an umbilical fascial incision is made. The Roux loop is mobilized to a length of 50–60 cm, with the distal end left free to allow for further mobilization while the proximal end remains fixed. The Roux limb is then brought up, and an end-to-side anastomosis is constructed. The intestine is returned to the abdominal cavity, and the umbilical defect is closed. Next, the Roux limb is transmesenterically positioned posterior to the transverse colon. A portoenterostomy is performed using separate 5/0 PDS sutures. Prophylactic drains are not routinely placed.

Representative operative steps are shown in Supplementary Video.

### Postoperative management

All patients were closely monitored in the neonatal intensive care unit (NICU) for the first 48 h postoperatively. Planned extubation was performed within six hours after surgery. Enteral feeding was initiated on postoperative day two, and steroid therapy with prednisolone at 4 mg/kg for the first three days, followed by a dose reduction of 1 mg every three days until reaching a final dosage of 0.5 mg/kg, was commenced on postoperative day three to reduce inflammation and support bile flow. Hormonal therapy was administered for a minimum of 15 days postoperatively.

Adjunctive therapy included the administration of antibiotics (meropenem), ursodeoxycholic acid, and fat-soluble vitamin supplements (Vitamins A, D, E, and K), along with oral calcium, in accordance with institutional protocol. The discharge criteria initially included the absence of surgical and infectious complications and the completion of the initial phase of steroid therapy. Subsequently, the criteria were expanded to include patient stabilization, with steroid therapy continued at the patient's place of residence.

Patients underwent regular clinical follow-up with serial liver function tests to monitor outcomes and detect any complications. Outpatient follow-up visits were scheduled one month after discharge and subsequently every three months.

In the studied cohort, 33 infants with biliary atresia were included and categorized into three groups based on age at the time of the LKPE procedure: under 60 days (*n* = 12), 60–89 days (*n* = 13), and 90 days or older (*n* = 8). The grouping was performed based on previously published studies demonstrating differences in prognosis depending on the timing of surgery. *post hoc* analysis showed that with the observed effect size (OR = 3.6), the sample size of 33 patients, and the given model parameters, the study had a statistical power of 64%. This means the ability to detect a true effect was moderate, and some non-significant results could be due to the limited number of patients rather than the absence of a real effect. The median age in these groups was 54.5 (27–58), 70 (60–89), and 99 (90–148) days, respectively (*p* < 0.001). The sex distribution did not differ statistically among the groups (*p* = 0.105). The most common type of biliary atresia was type III, diagnosed in 87.9% of patients, with no differences between groups (*p* = 0.246). Total bilirubin levels at admission were comparable across groups (*p* = 0.878), as were GGT levels (*p* = 0.413). All perioperative data are presented in Table 1.

**Figure 2:**
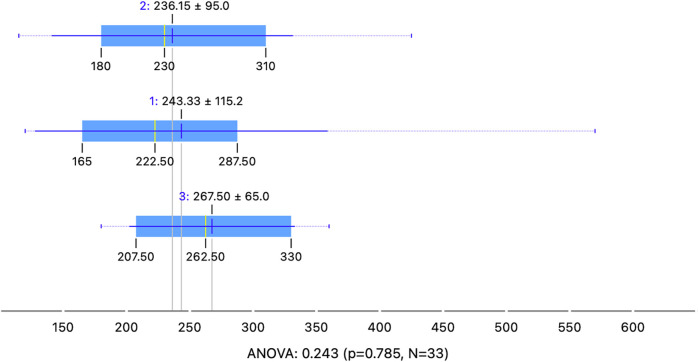
Comparison of operative time between groups. The analysis of variance did not reveal a statistically significant difference in operative time.

Analysis of the duration of the procedure showed that the median operative time in the group of infants under 60 days was 222.5 min (120–570), in the 60–89 days group was 230min (115–425), and in the group older than 90 days was 262.5min (180–360). However, the differences did not reach statistical significance (*p* = 0.785, Figure 2). Blood loss during the surgeries was minimal. In one case, a conversion from laparoscopic surgery to open surgery was performed (*p* = 0.478). The reason for the conversion was poor visualization of the hepatic hilum structures due to minor bleeding. The patient underwent an open Kasai procedure without intraoperative surgical complications.

**Figure 3:**
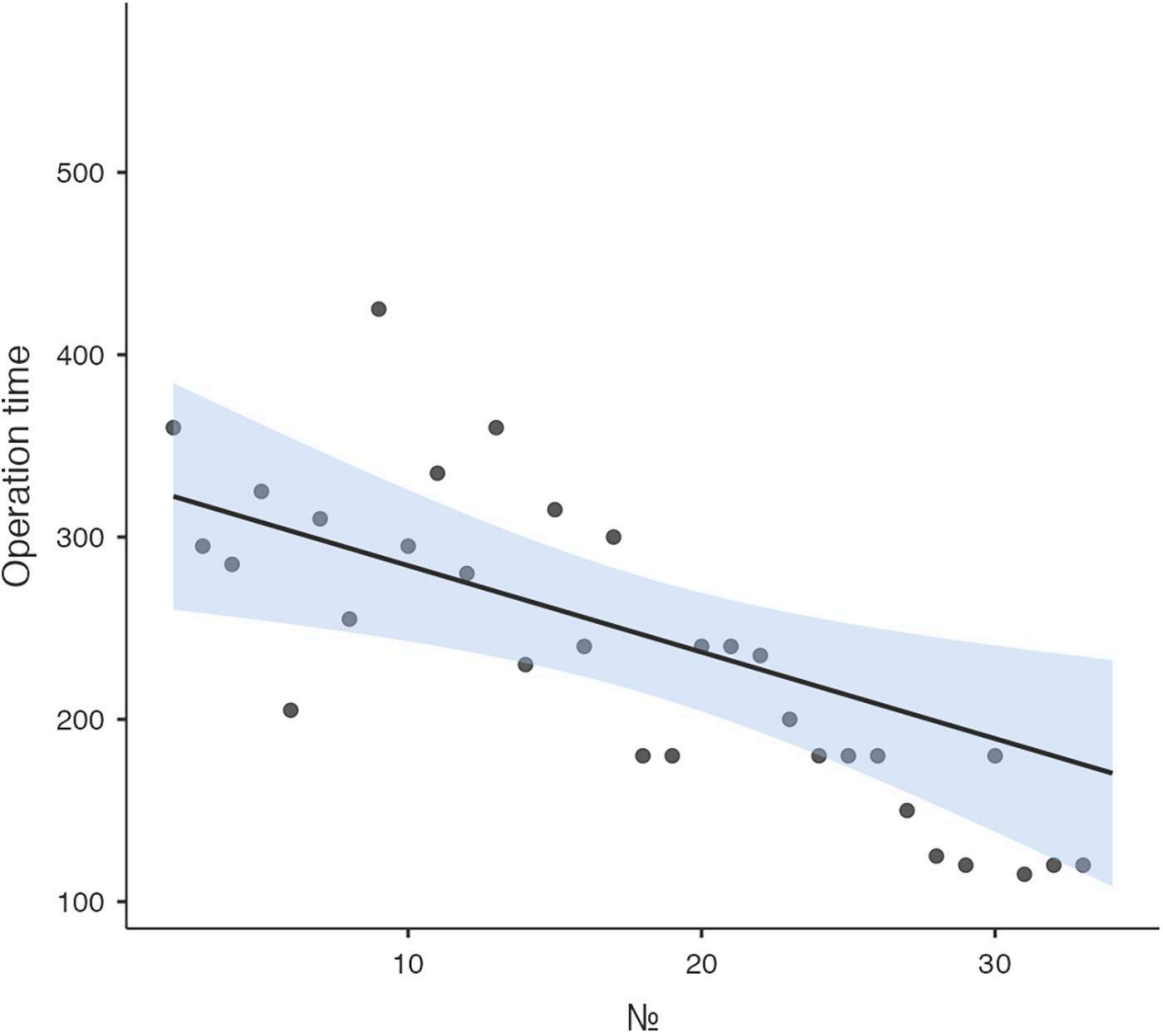
Learning curve analysis. Linear regression analysis of the learning curve demonstrated a reduction in operative time with increasing surgical expertise.

**Figure 4:**
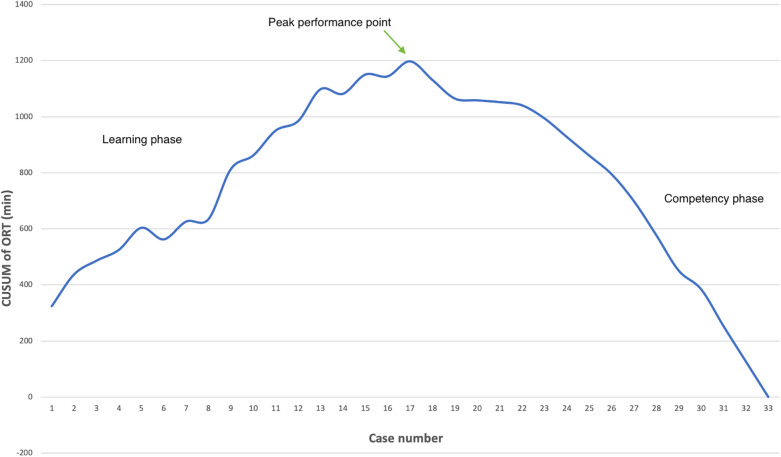
Learning curve analysis. The CUSUM chart illustrates the learning curve for laparoscopic Kasai procedures based on operative time. The upward trend during the initial 16 cases represents the learning phase, peaking at case 17 (annotated as the peak performance point), after which a plateau and subsequent decline indicate the transition to the competency phase. CUSUM was calculated as the cumulative deviation of each case's operative time from the overall mean. Success was defined as operative time ≤ mean operative time (240 min); failure was defined as operative time > 240 min.

**Figure 5:**
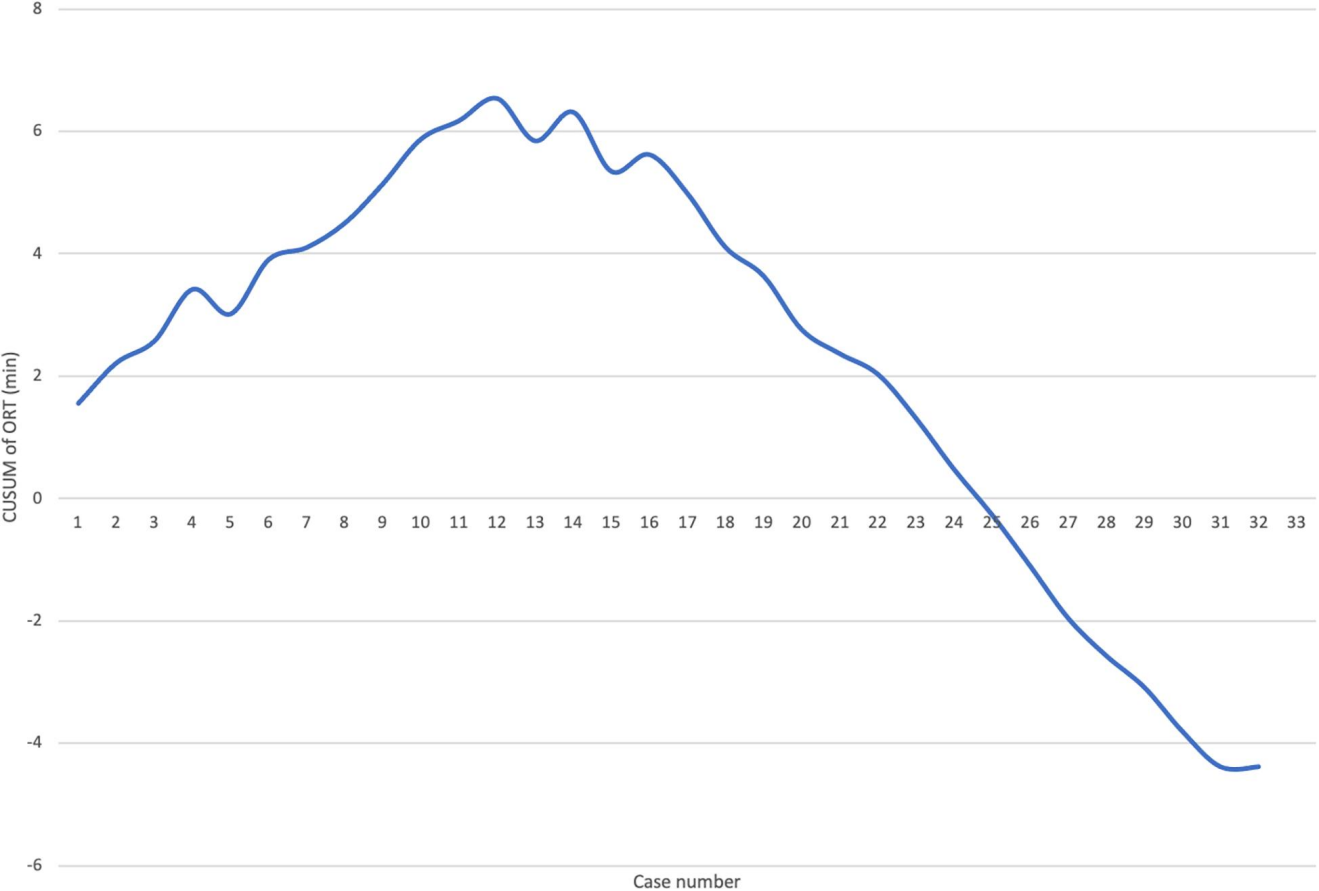
Learning curve analysis. RA-CUSUM learning curve for laparoscopic Kasai portoenterostomy based on operative time. The curve demonstrates an initial upward trend, peaking around the 13th–15th case, followed by a sustained decline, indicating improved performance and stabilization of the procedure. The RA-CUSUM accounts for individual patient risk factors derived from logistic regression modeling (including age, BA type, total bilirubin, and other covariates), offering a more precise depiction of the learning process.

Logistic regression analysis of the operative time revealed a reduction in procedure time with accumulating experience (R = 0.810, *R*^2^ = 0.656). However, the standard error of the model (59.2min) suggests the presence of additional factors influencing the duration of the procedure (Figure 3). CUSUM analysis demonstrated an initial increase in operative time, indicating the learning phase, with a peak observed around cases 16–17 (Figure 4). The curve was best modeled as a second-order polynomial with the equation: CUSUM (in minutes) = −0.0290 × (case number)^2^ + 0.7595 × (case number) + 0.3267 (*R*^2^ = 0.959). Risk-adjusted CUSUM analysis was additionally performed to account for patient-specific factors. The RA-CUSUM curve demonstrated an initial rise, peaking at approximately the 13th to 15th case, followed by a steady decline toward the end of the series (Figure 5). This pattern confirmed the existence of a distinct learning phase, after which operative performance stabilized and improved, even when adjusted for case complexity. Notably, the peak of the RA-CUSUM curve appeared earlier than in the standard CUSUM, suggesting that proficiency may have been achieved sooner than previously indicated. Following this, a stabilization and subsequent decline in operative time were noted, reflecting process optimization and accumulated surgical experience, ultimately leading to a reduction in operative duration.

As for the early postoperative period, no significant postoperative complications were observed in the patients. In one case, the patient developed severe hyperbilirubinemia (>600 µmol/L), which resolved spontaneously by the fifth postoperative day. Stool discoloration occurred in 97% of the patients after surgery. The only patient who did not experience stool discoloration after the surgery was the patient who underwent conversion. This was the second patient in our series, treated before we implemented standardized postoperative protocols for cholangitis prevention (including corticosteroids and prophylactic antibiotics). During surgery, we had to convert to an open approach because of dense adhesions and unclear hilar anatomy, which likely limited the complete removal of the fibrotic plate. In the early postoperative period, the patient developed cholangitis, which we believe led to the absence of stool color change, indicating persistent biliary obstruction. This patient passed away at home five months after the operation.

The median length of hospitalization was 14 (5–20) days and did not differ between the groups (*p* = 0.584). The incidence of cholangitis after surgery was 51.5% (*n* = 17), with no statistical difference between the groups (*p* = 0.631). Cholangitis developed at various time points after the surgery. A Cox regression analysis was conducted to evaluate the impact of cholangitis on native liver survival. Cholangitis was found to increase the probability of native liver loss by 3.6 times (*p* = 0.051). Multivariable logistic regression identified postoperative cholangitis as the only independent predictor of native liver failure (OR = 0.035; 95% CI: 0.003–0.441; *p* = 0.010). Other variables, including age, BA type, total bilirubin, and GGT, were not statistically significant (Table 2).

**Table 2:** Multivariable logistic regression analysis of risk factors for native liver survival.

Variable	OR	95% CI	*p*-value
BA type	0.191	0.009–3.939	0.283
Age (per day increase)	0.956	0.896–1.021	0.182
Total bilirubin (per μmol/L)	0.973	0.946–1.001	0.056
GGT (per unit increase)	0.999	0.997–1.002	0.633
Cholangitis after surgery	0.035	0.003–0.441	0.010
Conversion to open	∼0.000	0.000—NA	1.000^a^

^a^ The estimate for “Conversion to open” is unstable due to sparse data (*n* = 1 event).

OR, odds ratio; CI, confidence interval.

The native 1-year liver survival rate at the time of the study's conclusion was 54.5%, with no differences between the groups (*p* = 0.557). In the group under 60 days, native liver preservation was observed in 7 patients (58.3%), in the second group in 8 patients (61.5%), and in the third group in 3 patients (37.5%). Liver transplantation after the Kasai procedure was required in 5 patients (15.2%), and the transplantation rate did not depend on the age at the time of the initial surgical intervention (*p* = 0.577).

During the follow-up period, 10 of 33 patients died, corresponding to an overall mortality rate of 30.3% in the study cohort, with no differences between the groups (*p* = 0.5). In the group under 60 days, the mortality rate was 3 patients (25%), in the 60–89 days group it was 3 patients (23%), and in the group over 90 days, it was 4 patients (50%). Analysis of the causes of death in the overall cohort revealed that the most common causes were sepsis (4 cases, one of which was sepsis after liver transplantation), gastrointestinal bleeding (1 case), and aspiration (1 case). In the 60–89 days group, one patient developed acute renal failure, and another developed multiple organ failure. The cause of death in two patients could not be determined.

**Figure 6:**
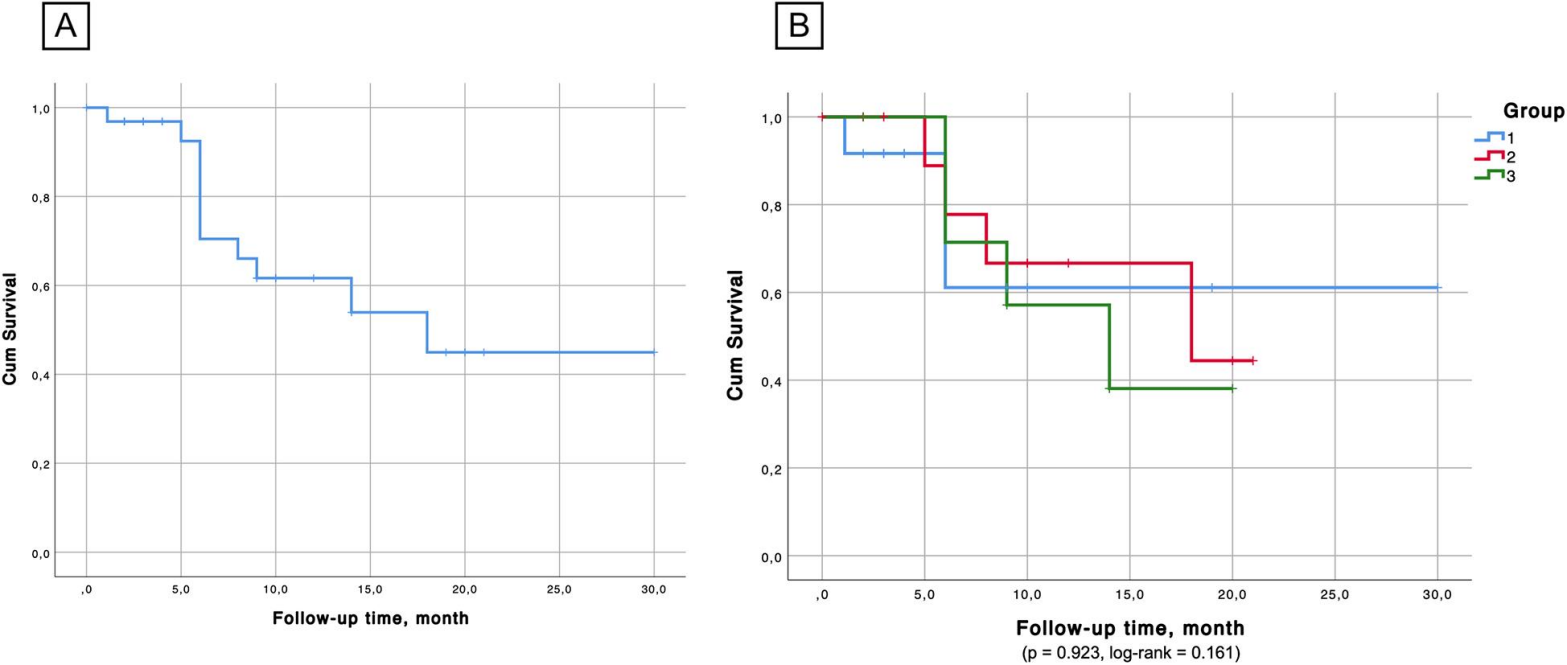
**(А)** Overall survival probability in the cohort. **(B)** Kaplan–Meier survival showed no significant differences between the groups in terms of survival probability.

**Figure 7:**
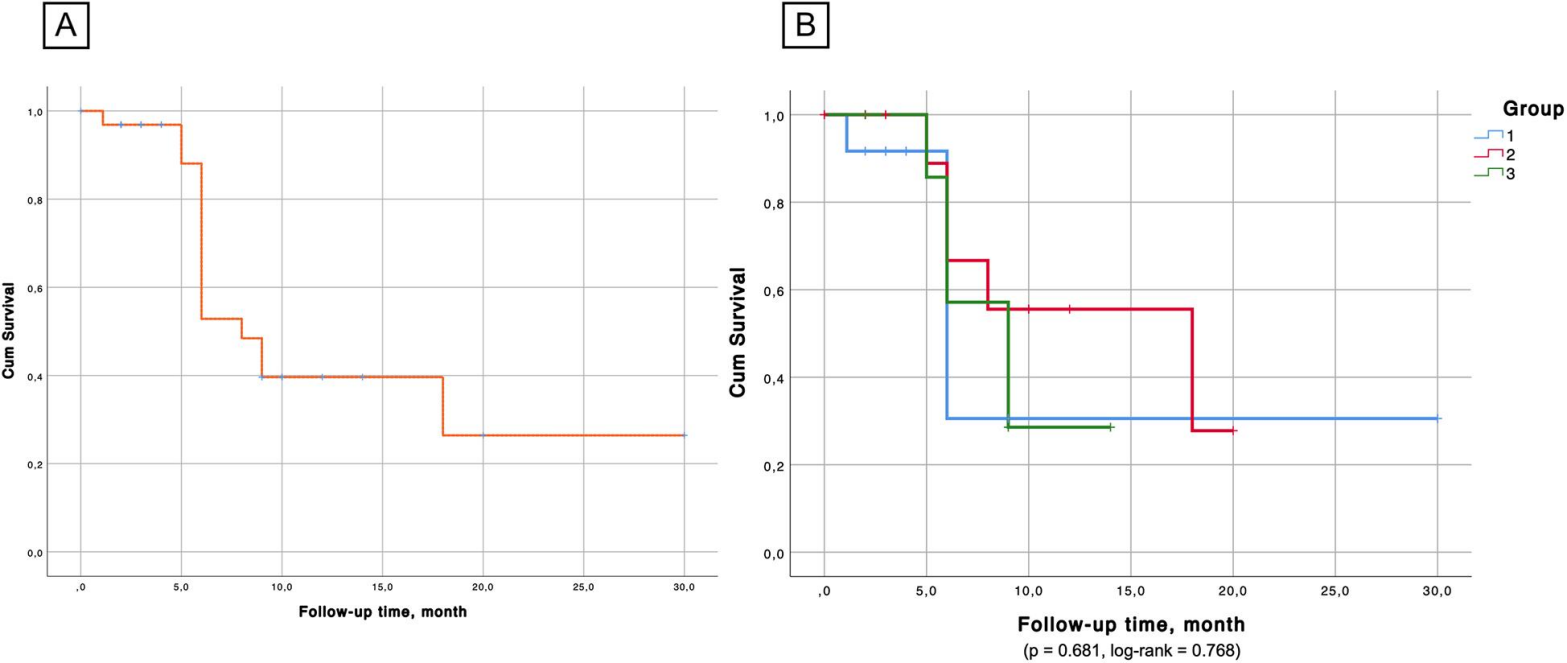
**(А)** Native liver survival probability. **(B)** Kaplan–Meier survival analysis confirmed the absence of significant differences between the groups in the aspect of native liver survival.

Kaplan–Meier survival analysis (Figures 6, 7) confirmed the absence of differences between the groups for both overall survival (*p* = 0.923, log-rank = 0.161) and native liver survival (*p* = 0.681, log-rank = 0.768).

Persistent jaundice was observed in two patients, who are currently being monitored on an outpatient basis and are awaiting liver transplantation. The remaining surviving patients achieved complete bile drainage with normalized liver function tests and no symptoms of jaundice.

## Discussion

LKPE has emerged as a minimally invasive alternative to the conventional open KPE for the management of BA (4). Our single-center study analyzed 33 cases of LKPE, providing insights into its feasibility, outcomes, and challenges. The findings suggest that while LKPE is technically demanding, it is a viable approach with favorable short-term outcomes, though certain challenges, particularly postoperative cholangitis, continue to impact native liver survival.

Our cohort consisted of 33 patients, which reflects the rarity of biliary atresia and the limited number of cases managed at our center during the study period. Although the sample size aligns with other single-center reports, *post hoc* power analysis demonstrated that the study was underpowered to detect subtle but potentially meaningful differences in outcomes, particularly across age subgroups. This limitation emphasizes the need for larger, multicenter trials to confirm the observed trends and strengthen the evidence base for laparoscopic Kasai portoenterostomy.

Our study demonstrated that LKPE is a feasible procedure, with only one case requiring conversion to open surgery due to inadequate hilar visualization. Our CUSUM analysis indicated that the learning curve plateaued after approximately 16–17 cases, suggesting a relatively rapid attainment of technical proficiency. The incorporation of RA-CUSUM analysis provided a more nuanced understanding of the surgical learning curve by adjusting for individual patient risk factors. While the traditional CUSUM curve demonstrated the expected learning pattern, the RA-CUSUM revealed that the peak of operative time deviation occurred earlier (around cases 13–15), indicating that skill acquisition may have been reached sooner than suggested by the unadjusted analysis. Moreover, the steady decline after the peak in the RA-CUSUM curve highlights sustained improvement in operative performance, even in more complex cases. This threshold is shorter than reported in previous studies: Ji et al. (8) suggested that stabilization required up to 30 cases, while Shirota et al. (5) noted plateauing at around 50 cases. We believe that several factors contributed to this difference. First, the procedure in our center was consistently performed by a single surgeon, ensuring continuity of technical refinement and minimizing inter-operator variability. Second, modifications to the surgical technique, including optimized trocar placement and the use of a 10 mm high-definition laparoscope, enhanced visualization and maneuverability, which likely contributed to improved operative efficiency. These adaptations may have facilitated a steeper initial learning curve, although differences in patient characteristics and institutional settings should also be considered when interpreting these results. The increased duration of surgery in the initial cases can be attributed to the technical complexity of hilar dissection and the construction of the Roux-en-Y anastomosis under laparoscopy. However, as experience accumulates, operative times become more comparable to those of open KPE (15–17).

We believe that several factors contributed to the reduction in operative time. Initially, we used a 5 mm camera but later transitioned to a 10 mm optic, which significantly improved visualization and enhanced the surgeon's comfort. Additionally, in the early phase of our experience, liver fixation was achieved via suspension of the round ligament; however, this approach did not provide adequate visualization of the hepatic hilum due to the overhanging segment IVb. Consequently, we transitioned to using a 3 mm instrument inserted percutaneously lateral to the hepatic projection, parallel to the hilum. This technique requires an additional assistant but significantly improves visualization of the hepatic hilum and reduces operative time.

In the present study, we observed an NLS rate of 54.5% and a patient survival rate of 84.8% following LKPE, with a median operative time of 240 min and a 3% conversion rate. This aligns with previously reported NLS rates following both LKPE and open KPE (7, 18). For instance, Shirota et al. (5) reported a 61% 1-year NLS among 53 patients with an operative time of ∼350 min and a ∼5% conversion rate, while Ji et al. (8) documented a 74% 1-year NLS with 100% patient survival across 100 cases, albeit with a higher conversion rate of 11%. Similarly, Li et al. (10) reported a 68% 1-year NLS and 96% patient survival in 49 patients. Other earlier studies, such as Wang et al. (19) and Chan et al. (20) reported 1-year NLS rates of 56% and 50%, respectively. However, despite the advantages of laparoscopic magnification, our study did not demonstrate a clear superiority of LKPE over open KPE in terms of survival outcomes compared with the literature (4, 6). One possible reason is the continued high incidence of cholangitis (51.5%), which remains a major determinant of postoperative morbidity. In our series, the cholangitis rate (51.5%) aligns with the broad range reported in the literature (12% to 66%), reflecting the persistent challenge of postoperative cholangitis despite technical refinements (21–27).

Table 3 summarizes the comparative outcomes of laparoscopic Kasai portoenterostomy reported in previous studies alongside our results (4, 5, 9, 28–44). Notably, while our median operative time (240 min) was shorter than that reported by Shirota et al. (5) (∼350 min) and Ji et al. (8) (274 min), it remains comparable to early reports by Wang et al. (16) (208 min) and Ure et al. (22) (166 min). We attribute our operative efficiency to technical modifications, as discussed earlier.

**Table 3:** Comparative outcomes of laparoscopic Kasai portoenterostomy.

First author	No of cases	Type of BA	Mean age at LapPE (days)	Mean operative time (mins)	Conversion rate Lap → Open (%)	Incidence of complications	SNL at 6 months old (%)	SNL ≥ 1 year old (%)
Current study	33	I: 1; IIa: 1; IIb: 2; III: 29	69 (median)	240 (median)	3	Cholangitis 17/33 (51.5%)	78	54.5
Sherota et al. (5)	53	NA	55	341	5	Cholangitis (43.%)	NA	61
Li et al. (9)	80	III	NA	NA	NA	Cholangitis 24/80 (30%)	NA	66.2
Nakamura et al. (28)	12	III	NL: 59.6	NA	NA	NA	95.2	79.6
Nakamura et al. (29)	36	MBD+	NNL: 81	63.8	52.7	NA	86.7	76.7
Nakamura et al. (30)	17	II: 1; MBD−; III: 16	66.7; 66.7; 65.5	NA	NA	NA	NA	76.5
Sun et al. (4)	48	NA	61.5	169.5	8.3	Persistent bleeding 4/48 (8.3%); Cholangitis 28/48 (59.09%)	81.82	78.12
Murase et al. (19)	12	NA	53	307.0	0.0	Cholangitis 1/12 (8.3%)	91.7	NA
Yamataka et al. (34)	15	NA	NA	NA	0.0	Cholangitis 7/15 (46.7%)	93.3	80.0
Wada et al. (24)	12	II: 1; III: 11	65.8	546.0	0.0	Cholangitis 6/12 (50%)	NA	83.3
Chan et al. (23)	16	III	65.6	NA	NA	Cholangitis 7/16 (43.7%); Variceal bleeding 1/16 (6.2%); Volvulus 1/16 (6.2%)	NA	50.0
Wang et al. (31)	25	II: 1; III: 24	72	208.0	0.0	Cholangitis 7/25 (28%)	NA	84.0
Yamataka et al. (20)	8	III	67.8	NA	0.0	Cholangitis 4/8 (50%)	87.5	75.0
Diao et al. (32)	4	I, II	24.4	126.0	0.0	0%	100.0	NA
Chan et al. (33)	16	III	65.6	NA	NA	Cholangitis 7/16 (43.7%); Variceal bleeding 1/16 (6.2%); Volvulus 1/16 (6.2%)	100.0	50.0
Oetzmann von Sochaczewski et al. (34)	8	NA	NA	NA	NA	Cholangitis 5/8 (63%)	NA	0.0
Yamataka et al. (11)	8	NA	67.8	NA	0.0	Cholangitis 4/8 (50%)	87.5	75.0
Koga et al. (10)	5	III	74	546.0	0.0	Cholangitis 1/5 (20%)	100.0	80.0
Chan et al. (35)	16	III	66	292.0	0.0	Cholangitis 3/16 (18.7%); Volvulus 1/16 (6.2%)	NA	56.2
Ure et al. (22)	12	NA	57	NA	0.0	0%	42.0	NA
Liem et al. (36)	11	II, III	79	245.0	0.0	Cholangitis 3/11 (27.2%)	NA	54.5
Liu et al. (37)	10	I: 6; II: 4	53	180.0	0.0	0%	NA	NA
Wong et al. (21)	9	NA	68.6	NA	0.0	Volvulus 2/9 (22%); Internal hernia 2/9 (22%)	33.0	22.0
Ayuso et al. (38)	5	NA	58	220.0	0.0	Volvulus 1/5 (20%)	NA	NA
Aspelund et al. (39)	5	NA	68.6	309.0	0.0	Wound infection 1/5 (20%); Cholangitis 1/5 (20%); Bile leakage 1/5 (20%)	60.0	NA
Dutta et al. (40)	7 (Lap) + 3 (Robot)	NA	NA	64.4	0.0	Wound infection 1/7	71.4	57.1
Lima et al. (41)	1	NA	90	NA	NA	0%	NA	NA
Lopez et al. (42)	1	NA	20	220.0	0.0	0%	100.0	100.0
Al-Qahtani et al. (43)	2	NA	45	450.0	0.0	0%	NA	NA
Martinez-Ferro et al. (6)	41	NA	61	180.0	2.4	Bleeding 1/41 (2.4%); Lt. HA transection 1/41 (2.4%)	NA	60.0
Martinez et al. (44)	3	NA	NA	220.0	NA	NA	NA	NA
Lee et al. (5)	2	III	98	338.0	0.0	Cholangitis 1/2 (50%)	50.0	NA
Esteves et al. (4)	2	NA	57	190.0	0.0	Cholangitis 1/2 (50%); Umbilical hernia 1/2 (50%)	100.0	NA

The Kaplan–Meier survival curve in our analysis estimates liver survival probability across the observation window, but further long-term follow-up is required to fully match the extended outcomes reported by other centers.

Taken together, these comparisons indicate that despite being conducted in a resource-limited setting, our center's outcomes for LKPE are largely consistent with international experiences, underscoring the procedure's technical feasibility and clinical relevance. The data also reinforce the importance of surgical experience and postoperative management in optimizing outcomes for biliary atresia.

Cholangitis remains a major postoperative concern, with an incidence of 51.5% in our cohort. This figure is within the reported range for both laparoscopic and open procedures (45). Our Cox regression analysis suggested that cholangitis increased the probability of native liver loss by 3.6 times, though statistical significance was borderline (*p* = 0.051). The logistic regression analysis confirmed that postoperative cholangitis significantly increased the risk of native liver failure (Table 2). This association remained robust after adjusting for age, BA type, and bilirubin levels, highlighting cholangitis as a major prognostic factor. The high incidence of cholangitis may be attributed to ascending bacterial infections, altered bile flow dynamics, and prolonged bile stasis postoperatively. Several strategies have been proposed to mitigate this risk, including routine antibiotic prophylaxis, early initiation of enteral feeding, and improved bile drainage techniques (18, 46). Of the first 10 cases of laparoscopic Kasai portoenterostomy (LKPE) performed at our center, seven patients succumbed within 6 months, primarily due to complications related to cholangitis and subsequent liver cirrhosis. This prompted us to modify our postoperative management strategy to reduce the incidence of postoperative cholangitis. To mitigate inflammation in the portal plate region and anastomosis, we introduced steroid therapy protocols immediately after surgery. Additionally, a prolonged antibiotic regimen was implemented, including trimethoprim-sulfamethoxazole (co-trimoxazole) for a minimum of 12 months postoperatively. Depending on clinical indications, the duration of co-trimoxazole therapy could be further extended. This approach led to a reduction in mortality and an improvement in native liver survival rates. Additionally, in the last several cases, we have utilized high-dose lactulose to promote improved stool passage and reduce the risk of intestinal stasis. This approach helps to lower the risk of ascending cholangitis in the early postoperative period. Future studies should focus on optimizing postoperative antibiotic regimens and exploring the role of adjunctive therapies such as ursodeoxycholic acid and probiotics in reducing the incidence of cholangitis.

The Kaplan–Meier analysis confirmed that survival rates did not significantly differ based on the age at surgery. While early surgery (<60 days) has traditionally been associated with better outcomes (47), our findings suggest that factors such as postoperative complications and the severity of the underlying disease at the time of surgery may also play crucial roles in determining long-term success.

A major challenge faced by our center is the lack of a well-established pediatric liver transplantation program. Currently, the Kasai procedure remains the only surgical option available for infants with BA, making its success critically important. In many developed countries, LKPE is seen as a bridge to liver transplantation, allowing infants to gain sufficient weight and optimize their condition before transplant (12, 48). However, in Uzbekistan, financial and logistical constraints make access to liver transplantation challenging, leading to high mortality rates in cases of failed Kasai procedures.

There are no precise statistics on the detection of biliary atresia in Uzbekistan. The primary healthcare system is underdeveloped, and as a result, patients often present either too late or pass away at their place of residence. The country lacks standardized protocols for the diagnostic evaluation of children with this condition.

Since the initiation of the LKPE program at our center, an average of five new cases of biliary atresia are referred to our facility each month. However, some of these patients present at an advanced stage when surgical intervention is no longer feasible. Others are referred abroad for liver transplantation. Currently, we are actively working to train healthcare providers in regional centers to improve the early detection of biliary atresia and ensure the timely referral of these critically ill patients to our center. Efforts are underway to develop a pediatric liver transplantation program in our country, which will be a crucial step in improving survival outcomes for infants with BA. Until then, it is imperative to refine our LKPE technique and optimize postoperative management to maximize the chances of long-term native liver survival.

This study is limited by its retrospective nature and relatively small sample size. Additionally, the follow-up period is still relatively short, and long-term survival data will be crucial to better understand the true efficacy of LKPE. Future research should include prospective multicenter studies with longer follow-ups to compare laparoscopic and open approaches in different clinical settings.

In addition to surgical refinements, future research should focus on optimizing postoperative management to reduce the incidence of ascending cholangitis, which remains a major contributor to native liver failure. Recent studies have explored extended antibiotic prophylaxis, the use of probiotics to stabilize gut microbiota, and the potential role of ursodeoxycholic acid in improving bile flow and reducing stasis (3, 46). Although these strategies show promise, high-quality randomized controlled trials are still lacking, particularly in resource-limited settings where liver transplantation is not readily available. Our findings highlight the need for a multidisciplinary approach that combines technical improvements with targeted medical therapies to optimize long-term outcomes after LKPE.

## Conclusion

Our findings suggest that LKPE is a feasible and effective alternative to open Kasai portoenterostomy, with comparable native liver survival rates according to literature and a clear learning curve. However, the high incidence of postoperative cholangitis remains a significant concern. In settings like Uzbekistan, where pediatric liver transplantation is not yet fully developed, optimizing surgical techniques and postoperative care for LKPE is of paramount importance. Further studies are needed to refine strategies for reducing cholangitis-related complications and improving native liver survival rates.

## Data Availability

The raw data supporting the conclusions of this article will be made available by the authors, without undue reservation.
